# Market mechanisms for newborn health in Nepal

**DOI:** 10.1186/s12884-017-1599-7

**Published:** 2017-12-19

**Authors:** Karsten Lunze, Rosie Dawkins, Abeezer Tapia, Sidharth Anand, Michael Chu, David E. Bloom

**Affiliations:** 1 Department of Global Health and Population, Harvard T.H. Chan School of Public Health, 677 Huntington Avenue, Boston, MA 02115 USA; 2 Boston Medical Center, 801 Massachusetts Avenue, Boston, MA 02118 USA; 3 Harvard Business School, Soldiers Field, Boston, MA 02163 USA

**Keywords:** Health technology, Social entrepreneurism, Incentive, Newborn, Hypothermia, Nepal

## Abstract

**Background:**

In Nepal, hypothermia is a major risk factor for newborn survival, but the country’s public health care sector has insufficient capacity to improve newborn survival given the burden imposed by distance to health facilities and cost. Low-cost technology to provide newborn thermal care in resource-limited environments exists, but lacks effective distribution channels.

This study aims to develop a private sector distribution model for dedicated newborn thermal care technology to ensure equitable access to thermal protection and ultimately improve newborn health in Nepal.

**Methods:**

We conducted a document analysis of newborn health policy in Nepal and a scoping literature review of approaches to newborn hypothermia in the region, followed by qualitative interviews with key stakeholders of newborn health in Nepal.

**Results:**

Current solutions addressing newborn hypothermia range from high-technology, high-cost incubators to low-cost behavioral interventions such as skin-to-skin care. However, none of these interventions  are currently implemented at scale. A distribution model that provides incentives for community health volunteers and existing public health services in Nepal can deliver existing low-cost infant warmers to disadvantaged mothers where and when needed. Newborn technology can serve as an adjunct to skin-to-skin care and potentially create demand for newborn care practices.

**Conclusion:**

Harnessing market forces could promote public health by raising awareness of newborn challenges, such as newborn hypothermia, and triggering demand for appropriate health technology and related health promotion behaviors. Market approaches to promoting public health have been somewhat neglected, especially in economically disadvantaged and vulnerable populations, and deserve greater attention in Nepal and other settings with limited public health service delivery capacity.

## Background

Despite significant efforts for children under 5 years of age, newborn survival has progressed insufficiently in Nepal. Although the country’s current newborn mortality rate decreased compared with 59/1000 live births in 1990, it remained stagnant at 33/1000 in 2006 and 2011 according to the Nepal Demographic and Health Surveys (DHS) [1]. Over the past two decades, the proportion of newborn deaths in Nepal contributing to overall child mortality even increased from 24 to 61% of all deaths in children under 5 years of age [2, 3]. Each year, more than 4700 newborns die in Nepal on their first day of life, and 13,000 die in their first 28 days of life [4], mostly due to problems related to prematurity (27% of newborn mortality), birth asphyxia (29%), or severe infections (24%) [3].

Newborn hypothermia, defined as an abnormally low body temperature below 36.5 °C [5], is an important contributor to these causes of death, particularly in high-risk environments such as Nepal [6]. It is associated with a significantly increased mortality risk [7], particularly in premature or low-birth-weight infants [8], half of whom globally are born in South Asia [9]. Newborn hypothermia is highly prevalent in Nepal: Even in institutions with skilled birth attendants, recorded prevalences range between 63 and 85% [10–13]. The most recent data from a community-based study in Nepal indicate that hypothermia is nearly universal [14]. While hypothermia risk is higher in the cold season, incidence in the hot season is also high [14]. Given a newborn’s physiological vulnerability to hypothermia, year-round thermal protection is required, even in warmer environments [6].

For thermoprotection of newborns, the World Health Organization (WHO) recommends a “warm chain” of heating the delivery room, immediate drying, providing skin-to-skin care, early and exclusive breastfeeding, postponing bathing, providing appropriate clothing and bedding, and placing mother and baby together [15]. However, implementing effective interventions such as skin-to-skin care has been challenging in many settings, particularly in Asia [16]. Because “warm chain” thermoprotective behaviors are often not practiced, health technologies have been proposed to facilitate the implementation of skin-to-skin care and other interventions [17]. One such technology purposely chosen for this study because of its innovative design and potential in resource-limited settings is a low-cost, sleeping bag–like infant warmer with a warming element designed for resource-limited environments [18]. Results from a clinical trial in Bangalore, India, suggest that the device achieves a statistically significant higher body temperature and effectively maintains body temperature compared with radiant warmers, blankets, and other methods to warm babies in hospitals with limited resources [19].

The WHO acknowledges that health technologies and medical devices are indispensable in health care delivery, but also recognizes that their affordability and appropriateness in low-income countries are still insufficient [20]. The WHO Global Initiative on Health Technologies, launched to encourage affordable innovation for resource-limited communities, found distribution challenges to be a major impediment to commercializing innovative, accessible, robust, and affordable health technologies for resource-limited environments [21].

Resource constraints and an unstable political system substantially limit adequate distribution of appropriate health technology in Nepal’s public health care sector. The aftermath of the April 2015 earthquake also added immensely to the Nepalese public health care system’s already overstretched burden. Nepal’s private sector has historically provided important health care capacity, but at relatively high costs to patients. In the absence of health insurance in Nepal [22], patients cover about 70% of total health costs out of pocket, totaling 3.6% of gross domestic product (GDP). While the private sector provides nearly twice the number of hospital beds of public hospitals, its profit-driven services are concentrated in the wealthy and urban areas of the country’s central region and do not reach economically disadvantaged rural populations [23]. Because Nepal’s disadvantaged are less able to pay out of pocket for private sector health services, they predominantly rely on public health services that now more than ever are inadequately resourced to meet demands [24].

Indeed, existing distribution channels for any health technology hardly extend beyond the capital city of Kathmandu and do not reach economically disadvantaged populations in remote rural areas. Traditionally, most public health interventions in resource-limited countries, including distribution of health technologies, substantially depend on limited public budgets or external, unsustainable grant funding.

To address the disadvantages of low-income populations that live remotely from health system structures, we developed a private sector device distribution model that takes on newborn hypothermia and ultimately newborn health in Nepal and ensures equitable access to newborn thermal protection across population income strata.

## Methods

This was a qualitative, formative study to develop a potentially self-sustaining implementation and distribution model for newborn health technology independent of conventional public or philanthropic funding. We did not aim to demonstrate effectiveness or efficacy of any particular device, but rather aimed to provide a concept for implementing a thermoprotective intervention for newborns in Nepal’s rural communities currently inadequately served by the public health system.

### Data collection

We systematically searched key policy documents and peer-reviewed literature in standard reference databases and through expert referral to assess the regional burden of newborn hypothermia and existing approaches to address it in Nepal. We also conducted qualitative in-depth interviews with key stakeholders in urban, suburban, and rural settings in and around Kathmandu and Narayangarh, Nepal, representing the fields of newborn health and social entrepreneurship. We identified informants through expert referral. Interviews followed a semi-structured questionnaire, soliciting information and probing attitudes about newborn care and hypothermia.

Using a purposive expert sampling strategy, we sampled a total of 21 potentially information-rich experts among Nepalese and international nongovernmental organizations (NGOs) (Nepal Family Health Program, *n* = 2; Mother and Infant Research Activities, n = 2; Nick Simons Institute, *n* = 1; and Save the Children, Kathmandu, n = 2), international organizations (the United Nations Children’s Fund [UNICEF], Kathmandu, n = 2), and social entrepreneurs (Ashoka Foundation, Nepal, n = 1). Further information was obtained from providers at urban hospitals (Kanti Children’s Hospital, n = 2, and Patan Hospital, Kathmandu, n = 2), from employed and volunteer health workers in rural health posts at various distances from urban centers (*n* = 4), and from rural educational facilities (Balkumari College, Narayangarh, *n* = 3). Based on respondents’ preferences, we conducted interviews in English or Nepali with simultaneous translation into English and transcribed pertinent quotes in English. We did not collect data on respondents’ identities and did not record any personal information. We informed all participants of the study purpose and obtained their verbal consent to participate before starting the interviews.

### Data analysis

We conducted a content analysis according to Miles, Huberman, and Saldaña’s approach to memoing and coding [25]. First, following a thematic analysis of key policy documents, we produced theoretical memos based on an initial interview data read to guide further data collection and iterative analysis. Then, in an initial cycle of content analysis, we manually coded responses into these categories: (i) local health care systems and their key stakeholders, (ii) health care practices and financing, and (iii) perceptions of available and needed services. To develop our theoretical model, we second-cycle coded and organized data by topic area such as health care system and newborn care, income generation, and savings groups and microfinance activities.

### Study setting

Nepal is one of the world’s most economically disadvantaged and least developed countries. More than 25% of its nearly 31 million people live below the poverty line, and the estimated 2015 GDP per capita was US$2500 in purchasing power parity (PPP), ranking 197 out of 230 countries globally [26]. After years of violent political struggle, the culturally distinct country is currently the youngest republic in the world and is transitioning from monarchy to democracy. Improvements in health have been prioritized as key to the still unfinished peace-building process in Nepal [27]. The country’s current Gini index of 32.8 has been decreasing, which indicates an increasingly equitable society [26], but substantial inequality still exists in post-conflict Nepal.

Nepal is geographically diverse and divided into distinct ecological zones. Environmental temperatures vary in the Terai lowlands and in the hill areas between slightly below 0° and 43 °C and in the mountain areas between far below freezing and 29 °C [2].

More than 1500 deliveries are estimated to take place in Nepal daily [4]. Most deliveries occur at home (63%), while 35% take place in a health facility (26% in public sector health facilities, 2% in nongovernment facilities, and 7% in private facilities) [1]. Delivery in a health facility varies widely by ecological region, is twice as common in urban areas, and is strongly positively associated with mother’s education and wealth quintile [1].

## Results

This section presents a scoping review of the literature and of Nepal’s policy documents pertaining to newborn children. It also sets forth our model for distributing thermoprotective technologies for newborns.

### Document and literature analysis

#### Newborn health in the context of the Nepalese health care system

Historically, Nepal enjoyed relative stability, though massive inequality, under a succession of monarchs until it entered into a violent struggle for democracy in the twentieth century. Since democracy was introduced in 1990, Nepal has experienced ongoing political instability. A decade of deprivation and violence during the Maoist insurgency beginning in 1996 negatively affected communities and basic health service delivery, particularly for women and children [28]. Although the Maoist revolution ended in 2006 with the declaration of Nepal as a federal democratic republic and an interim constitution came into force in 2007, the country remains politically unstable. Even before the 2015 earthquake, the post-conflict Nepalese health system suffered from lack of funds, chronic shortage of human resources, corruption, and inequitable access to health care [29].

Over 15 years ago, national researchers compiled the *2001 Situation Analysis Report* on newborn mortality, newborn health, and health care in the country [*cited in* 30]. In response, Nepal’s Ministry of Health and Population (MoHP) and NGOs such as Save the Children/Saving Newborn Lives created a Newborn Working Group to help develop a national newborn health policy. At that time, Nepal was the first Asian country to make newborn survival a development priority through the *Nepal Neonatal Health Strategy*, issued by the MoHP in 2004. To implement the policy, the MoHP conducted a rapid assessment of neonatal health programs in 2007 and, under the leadership of the Family Health and Child Health Divisions, engaged stakeholders to develop the *National Neonatal Long Term Plan* 2005–2017 [30].

This approach made newborn health a national priority. Since then, all policies in Nepal, such as the national periodic health plans (9th plan, 10th plan, and interim plan) and the *Nepal Health Sector Plans I* (2004–2009) and II (2010–2015), recognize newborn health as a component of essential health care services in Nepal and emphasize improved access for economically disadvantaged and vulnerable groups [31]. As a result, existing policies and strategies guided by the *Nepal Neonatal Health Strategy* provide both a conducive environment and a framework to develop and implement newborn innovations for disadvantaged people in the country [31, 32].

Nepal has four national hospitals, four regional and sub-regional hospitals, eight zonal hospitals, and 65 district hospitals, receiving referrals from 168 primary health care centers (which have a doctor, four nurses, and a goal of 15 beds including three maternity beds). Beyond the primary health care centers are 696 health posts providing antenatal and postnatal care—some with birthing centers. The more than 3100 sub-health posts also provide antenatal and postnatal care [1].

While the Nepalese public health care system theoretically provides health care and medications free of cost, in reality the system encounters significant resource limitations. Public health expenditure in Nepal is only 2.4% of GDP, or 5.8% of government expenditure, with an annual budget of about US$3 per capita [33]. Many urban but particularly rural populations in Nepal have no physical access to these services, cannot afford transportation costs, or lack knowledge about how to gain access. NGOs and private health care operate on a fee-for-service basis. Although several central public hospitals provide tertiary care without charge, these higher-level institutions are likewise insufficiently resourced.

Nepal’s health system relies heavily on its unique cadre of community health workers, the female community health volunteers (FCHVs). Almost 50,000 FCHVs, in general one per ward covering about 50 households, have been trained to serve the health care needs of the population at the community level [34]. FCHVs, many of whom are literate, are selected from women in the community [28]. In general, FCHVs spend an average of about 5 hours a week on primary care and prevention activities such as vaccination campaigns or community-based treatment of pneumonia and attend an average of about 16 deliveries per year [35]. Under the Nepalese MoHP’s newborn care package, FCHVs are trained in basic newborn care, and national policy aims for skilled birth attendants capable of attending both mother and child to attend all births [36].

### The impact of significant financial barriers on safe delivery practices in Nepal

In Nepal, most births (63%) take place at home, and skilled attendants assist only 36% [2]. High-risk delivery and hazardous newborn care practices at home, often rooted in traditional beliefs, remain common [37]. Even with trained birth attendants, beneficial thermoprotective practices are often neglected. Heating the birthplace, a first critical step for home birth, is not done consistently [38, 39]. Although wrapping the child and delayed bathing prevents heat loss from evaporation, most trained birth attendants simply place the baby on the floor after birth, often without drying or wrapping the baby, and many bathe the baby soon after birth [38–40].

A survey study from Nepal [41] found that the average household cost of a home delivery ranges from Nepalese rupees (NPR) 410 without a skilled birth attendant to NPR 879 with a birth attendant (US$5.85 to 12.56, respectively, at a rate of NPR 70 per US$ at the time of that study). An uncomplicated, facility-based delivery averaged fees of NPR 678 (US$9.70), with additional charges and opportunity and transport costs adding up to a total exceeding NPR 5300 (US$75), and a caesarean section averaged fees of NPR 11,400 (US$160). Based on these figures, the cost of financing current practice is NPR 45 (US$0.64) per capita. The per capita cost of universal institutional delivery is substantially larger than the cost of universal skilled birth attendance at home (NPR 400 vs. 117, or US$5.75 vs. 1.70, respectively) [41].

In light of the substantial household costs associated with childbirth, Nepal’s government developed and implemented the Safe Delivery Incentive Program in 2006 to motivate women to seek birth attendance at health institutions. This conditional cash transfer program offered incentives ranging from NPR 1500 (US$21.45) in the mountain regions to NPR 1000 (US$14.30) in the hills and NPR 500 (US$7.15) in the Terai lowlands, primarily to defray transportation costs to reach birthing facilities. Skilled birth attendants were to be paid NPR 300 (US$4.30) per delivery [42]. While financial incentives offered directly to the targeted population in the form of conditional cash transfer programs have had success in Latin America [43–45], Nepal’s *Safe Delivery Incentive Program* had considerable bureaucratic and communication problems [46].

### Low-cost interventions for resource-limited populations

To develop a newborn thermal care strategy for Nepal, we conducted a scoping review of newborn care and dedicated newborn health technology in a global context.

More than 2.2 billion people worldwide currently live at a poverty level of less than US$2 a day [47]. Newborn care interventions tend to be low-cost, but highly cost-effective, especially when integrated into existing delivery services. Globally, a package of interventions and scale-up of existing services to nearly universal coverage in the developing world could avert up to 72% of newborn deaths, which translates into almost 3 million lives saved [48]. This would cost an additional US$4.1 billion globally, or US$2100 per newborn death averted, beyond the current expenditures of US$2.0 billion for newborn survival [49]. Currently, newborn care interventions have not achieved scale or permanence globally, and local strategies are urgently required.

Implementation of culturally adapted methods of thermal care through continuous skin-to-skin contact, a key part of kangaroo mother care, is associated with a decreased infectious morbidity [50] and mortality [51] among low-birth-weight infants. However, no clear evidence shows that this strategy is sufficient to improve infant mortality outside of hospital settings, and it remains unavailable at scale in most resource-limited countries [51–53]. Furthermore, given various contextual barriers, implementation of skin-to-skin care has been challenging in many places, including Nepal.

To provide additional heat sources for effective thermal protection of newborns in resource-limited environments, academia and industry are developing various low-cost, low-technology newborn warming devices [18]. Both barriers to heat loss and external heat sources have been shown to be effective in reducing heat loss in infants [8]. One low-technology device, an infant warmer in a sleeping bag design incorporating a warming element, provides passive and active thermoprotection and was designed to complement or replace other available options for thermoprotection (Table 1) [47]. While the device provides a potential approach to the newborn hypothermia challenge, at present it lacks a distribution strategy and remains out of reach for most of Nepal’s population.

**Table 1:** Options for newborn thermal protection in resource-limited settings

Method	Eligibility	Advantages	Risks or disadvantages	Availability
**Skin-to-skin contact (SSC) by mother or other caregiver**	Stable infants	Mother can closely monitor, promotes bonding	Not for life-threatening conditions	Community and institutional
**Radiant warmer or water mattress**	At-risk infants	Allows observation of baby Allows procedures to be performed	Hyperthermia, dehydration Expensive to buy, requires electricity	Institutional only
**Incubator**	At-risk infants, including high-risk, low-birth-weight infants	Maintains constant temperature and humidity Easy provision of oxygen Allows observation of (naked) infant	Hyperthermia, dehydration Expensive to buy, maintain, clean; requires electricity Separation of mother and child	Institutional only
**Warm room**	Stable infants	No newborn equipment necessary	Hypothermia Uncomfortable for caregivers	Community and institutional
**Low-cost warming device (together with SSC)**	At-risk and stable infants	Mother can closely monitor, other caregiver can provide SSC	Not appropriate for life-threatening conditions; more expensive than SSC	Potentially community and institutional

### Newborn technology distribution model

Our scoping literature review and qualitative interviews provide data to develop a distribution model for this or similar newborn technologies. Our analysis of qualitative data identified relevant market segments and distribution strategies in Nepal as follows:

### Economically disadvantaged rural populations

Approximately 25% of the Nepalese population earns less than US$1 per day and has no disposable income beyond subsistence [26]. Many women in rural Nepal are organized in mothers groups with a strong community spirit. In general, much of the income-generating activity takes place in a communal context. Due to the limitations that economically disadvantaged families have in providing thermal protection for their newborns, the greatest burden of newborn mortality attributable to hypothermia falls on economically disadvantaged rural populations and makes access to newborn interventions a priority for this segment.

### Economically disadvantaged urban populations

Many economically disadvantaged rural individuals migrate to urban areas in search of work, where community ties are weaker than in rural areas. Although many economically disadvantaged urban people may not have much more disposable income than their rural counterparts, they do have better access to the public health system.

### High income

High-income populations in Nepal are willing to spend significant amounts for newborn care. This population stratum may find a device dedicated specifically to newborn thermal protection a cost-effective alternative to heating the home.

**Fig. 1:**
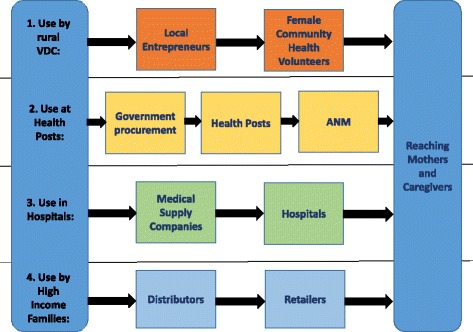
Business model overview: The intervention can be launched by manufacturers or through a wholesaler (ANM, auxiliary nurse midwife; VDC, village development committees)

Based on this market segmentation, we developed a four-pronged approach that includes local entrepreneurs to distribute the thermal protection device on a commercial basis (Fig. 1). The typical practice of distribution through businesses to private and public health care providers does not reach mothers and their infants directly. In addition, costs associated with any device make it unaffordable for most of the target population: economically disadvantaged rural and urban people. Our model, in contrast, involves social entrepreneurs and microfinance organizations to reach mothers and their newborns at the time and place of delivery, makes the device affordable to the economically most disadvantaged, and enhances the intervention’s sustainability. It also provides the economically disadvantaged a previously unavailable choice in how to use their limited resources, both individually and collectively.

We explored acceptable price ranges for different target groups among key informants. Suggested retail prices vary by segment and will eventually follow market dynamics, given the expected interaction among the segments. Marketed differentially, motivated wealthy urban buyers can afford and are expected to accept higher prices than economically disadvantaged urban or rural people. As a result of a cross-subsidization scheme at the distributor level, those with more purchasing power will make the intervention affordable for the most economically disadvantaged.

### Economically disadvantaged rural populations at the community level

In rural areas, about 50–120 households are grouped into wards, of which Nepal has about 35,000. In turn, wards are organized into about 3900 village development committees (VDCs). Rural VDCs represent the majority of the population and its most vulnerable families. Almost all mothers in rural villages live at subsistence level. Because individuals are too poor to pay for an intervention, our model targets mothers groups, of which about 50,000 exist nationwide, and Nepal’s numerous savings and microfinance groups. These groups usually pool NPR 5–500 (currently about US$0.05–4.70) per participant per month into a general fund. Another option is to target the 13,000 forest user groups—agricultural microcredit groups that include almost a third of Nepal’s population. Even though their activities are typically not directly related to health interventions, they do generate communal income.

Our model proposes to distribute the device to local entrepreneurs. To promote his or her commercial activities, an entrepreneur typically has relationships with specific community decision makers in a given territory. By virtue of their ability to mobilize savings, microfinance organizations then purchase the device directly from an entrepreneur. In a variation of this model, a forest user group could loan or donate funds to a mothers group to obtain a device. The mothers group in turn identifies an overseer of the device in the community to ensure its use at the time and place of a delivery.

In this rural distribution strategy, nationally active FCHVs potentially play a crucial role. Under the *Nepal Neonatal Health Strategy*, FCHVs deliver the *Community-Based Newborn Care Package*, a program to prevent and manage newborn infections, hypothermia, and asphyxia, and to develop an effective system for referral of sick newborns [54, 55]. Serving as the liaison to the entrepreneur to acquire the device; later assisting eligible mothers to allocate, use, and clean the device; and facilitating its acceptance by families, the FCHV would receive monetary compensation from the entrepreneur for her services. Thus, both the entrepreneur and the FCHV would have an incentive to distribute the device and, assuming its use is beneficial and culturally accepted, encourage its proper and sustainable distribution and use. A suggested price of NPR 3000 (currently about US$28) would be affordable for a savings group and would cover production costs and generate income for the entrepreneur and the FCHV, under the condition that policy makers and the community accept this as appropriate.

### Economically disadvantaged rural people at health posts

The government of Nepal publicly funds and provides all products and services at health posts. Although a minority of deliveries occurs at health posts, national policy aims to increase the proportion of institutional births. Typically, a health post with an average of two attended births per month should stock more than one device to protect the newborn until a device from the community is available for transport and use at home. Alternatively, a health post could loan the device to the mother for a deposit to ensure return of the device and possibly encourage the mother to make a follow-up visit.

The distribution of 8800 devices needed nationwide could occur through established government channels, which currently distribute other medical supplies to the health posts. A suggested price of NPR 6000 (US$56) per device was determined as acceptable in this segment. The total cost to the government of about US$ 493,000 corresponds to less than US$0.02 per capita (about NPR 2), which we consider a low-cost proposition for potentially life-saving thermal protection of newborns.

### Central and district hospitals

Like many developing countries, Nepal has an unmet need for secondary and tertiary care equipment in general and for thermal protection devices in particular. The need for incubators is neither met in better-equipped central hospitals in Kathmandu nor in district hospitals. On an institutional level, thermal protection is needed at the place of delivery, during transport to the nursery, in the nursery, and eventually at home to allow for early discharge. The newborn warming device meets these needs, with the exception of a small number of very high-risk, high-morbidity newborns requiring more specialized care.

At a suggested price of NPR 5000 (US$46.60) per device sold to hospitals through established medical products distribution companies, hospitals can stratify their distribution based on the income level of the patient:

I. To upper-middle-income or upper-income patients, provide the device to patients’ families upon discharge at the price of NPR 6000 (US$58) with a margin of NPR 1000 (US$9.30). Private hospitals can sell this device directly. Public hospitals may not sell medical supplies directly but can induce purchase of the device at nearby local pharmacy retail stores.
II. Rent the device out on a daily basis and for a deposit to ensure return of the device.
III. Loan the device for free to those unable to afford renting the device, also with collateral. Hospitals can make their own arrangements in this regard and may find early discharge of patients with the device beneficial, even if a deposit cannot be secured.

The potential target institutions span public district hospitals, primary health care centers, health posts, sub-health posts, and private sector hospitals, each with an estimated demand of 10–30 devices per year.

### Upper-middle- to high-income urban citizens

Upper-middle- to high-income urban families with newborns have better access to newborn health care. However, even private health care facilities do not provide adequate thermal protection. Another advantage of targeting this segment is the cross-subsidization of the costs of the other channels, particularly of savings and credit groups, thus making the device more accessible to economically disadvantaged rural populations. This product would be differentiated as a high-end product, with distinct material and packaging, different color and design options, and various sizes, to justify the substantially higher retail price compared with the rural market segment. The suggested target price of NPR 7000 (US$65) includes the marginal cost for the more elaborate production and a margin for distributors and retailers.

## Discussion

We present a concept of providing access to thermoprotective newborn technology for all segments of society in Nepal. The model specifically targets rural families in Nepal through social entrepreneurs, savings groups, and microcredit organizations to implement a newborn health intervention and distribute health technology to economically disadvantaged urban and rural populations that could otherwise not afford it at the time and place of the highest hypothermia burden. Our findings intend to inform planning, implementation, and dissemination of newborn health care interventions and health technology in the context of poverty and limited health infrastructure.

Appropriate technology for newborn care in primary and secondary health care facilities might be an important adjunct to community-based management strategies [50]. Traditionally, private sector providers have played an important role in fostering maternal and child health interventions, but were not necessarily structured for scale or permanence. The public sector, however, could potentially provide large-scale interventions, but has been weak with regard to efficacy and efficiency. In a changing global health landscape, both sectors must contribute to address the global burden of disease that falls disproportionally on low-income populations [56].

Founded on viable, profitable enterprises, business methods tailored to the economically disadvantaged could link scale and sustainability with efficacy and efficiency. Few markets are as highly motivated and attuned to innovative commodities and technologies as expectant mothers and their families are. Given that background, the business sector, with its power to innovate and ability to create sustainable models for constructing, deploying, and delivering products, might have a unique capacity to provide appropriate tools and technologies even in the remotest areas [57].

In the past 5 years, the government of Nepal introduced several newborn health programs, including the *Community-Based Newborn Care Package*, which has a birth-preparedness package, chlorhexidine application to a newborn’s umbilical cord to prevent infections, and the newborn vitamin A supplementation program to reduce morbidity and mortality during early infancy. These pilot programs have not been fully implemented or scaled up [58]. The 2015 earthquake, which added an immense burden to Nepal’s already strained public health care system, widened the delivery gap for economically disadvantaged rural and urban populations. Social enterprises and social marketing, the application of commercial marketing concepts and techniques to promote voluntary behavior change [59], might provide a missing link to deliver essential public health services or health technology to populations with limited income.

Selling a device to mothers groups or community income–generating groups that in turn sell or lend it to individual mothers might seem absurd given the poverty of most mothers in Nepal. However, experiences from various savings-led microfinance enterprises in South America and Southeast Asia suggest that profitable enterprises specializing in serving the low-income sector might have a measurable impact on the health of the economically disadvantaged [60]. By improving the health and thus the productive capacity of these people to a meaningful extent, commercial ventures addressing health might strengthen the ability of low-income populations to maximize their earnings and increase their assets, thereby improving quality of life for current and future generations [61]. Private sector organizations have developed several innovations in health service delivery with the potential to better serve the health needs of economically disadvantaged populations [62]. If for-profit health care proves profitable, these investments might be a step for developing countries toward escaping dependency on foreign aid [63].

Notably, our theoretical model does not exclude the public sector and its advantages in advancing health technology. For institutions and in urban areas, selling the device—marketed and priced differentially to health care institutions and to urban high-income users—might help to target it to low-income families through cross-subsidization.

In rural areas, this health technology delivery model is based on the services of an existing network of trained FHCVs who already have access to mothers and can thus provide effective thermal protection for infants, when and where these births occur. Given that national policy supports institutional births, the role of FHCVs in deliveries is controversial. However, FCHVs—who are trained in antenatal counseling and providing postnatal visits—have achieved notable improvements in household practices and service utilization [64]. Trained birth attendants in Nepal practice beneficial newborn interventions but are reported to neglect thermal care [40], a situation that our model specifically seeks to address. In distributing a device, the potential also exists for the FHCV to relay important educational messages to mothers about protecting their newborns from hypothermia. While limited literature exists on the quality of care they provide, FCHVs with higher education levels (secondary and above) and those who are active in areas where research programs and NGOs are already active have been found to perform satisfactorily [65].

This distribution model could also be aligned with or integrated into other, currently existing relevant activities, such as newborn resuscitation programs [66]. Participatory community-based interventions in women’s groups have had beneficial effects on home care, health care–seeking behavior, and newborn mortality outcomes [67]. Health technology can best innovate in the context of innovative approaches across the health systems building blocks, such as novel approaches to community ownership and participation, but also innovations regarding the health workforce and health financing [68].

Given the failure of conditional cash transfer programs in Nepal to increase institutional deliveries, FCHVs and other skilled or traditional birth attendants might bridge the gap to safe delivery practices where most births presently take place—at home. Providing financial incentives to volunteers, as our model proposes, does not necessarily conflict with volunteerism. Financial and non-financial incentives, designed in their given context to enhance intrinsic motivation, have been found essential to the maintenance and sustainability of community-based programs and proven to increase workers’ retention [69]. While microcredit groups may not always reach the poorest of the communities [70], newborn and maternal health aspects are meaningful microfinance activities, because mothers will take ownership. At the same time, our model integrates several innovations shown to improve the availability and affordability of health care for the economically disadvantaged, such as cross-subsidies, high-volume and low-cost models, and technical simplifications that focus on a circumscriptive clinical issue [71].

### Limitations

This study developed a distribution model for newborn health technology. Future studies will have to test its implementation and whether the device and the implementation of this model reduce newborn mortality due to hypothermia. Circumstances have clearly changed since the devastating 2015 earthquake, with income generation, infrastructure repair, and food security gaining prominence. Nevertheless, newborn survival remains a top priority in Nepal, providing strong support for the model’s continuing applicability.

## Conclusions

Newborn hypothermia is a major risk factor for newborn survival in Nepal, and resource limitations and distribution challenges are major impediments for most of Nepal’s population to access health technologies addressing this problem. Market-oriented incentives for an existing network of female community health volunteers and social entrepreneurs in Nepal might deliver newborn health interventions to economically disadvantaged mothers at the time and place of birth, while triggering demand for appropriate health technology and related health promotion behaviors.

Harnessing market forces could promote public health by raising awareness for newborn challenges such as newborn hypothermia. Based on commercial methods, this model’s implementation could potentially provide low-cost, low-technology thermal protection for newborns in Nepal in a timely manner, targeting specifically the poorest families. Application and evaluation of this model will further contribute to our understanding whether market mechanisms can promote the delivery of public health interventions to reach vulnerable populations effectively and efficiently.

Business methods tailored to economically disadvantaged populations could make interventions available at scale and make them sustainable, independent of external grant funding. Targeting low-income populations through local social entrepreneurship might bridge a gap between health service and health technology delivery and finally reduce disease burden. Market approaches to promoting public health have been somewhat neglected, especially for economically disadvantaged and vulnerable populations, and deserve greater attention in Nepal and other settings with limited public health service delivery capacity.

## Abbreviations

ANM: auxiliary nurse midwife
DHS: Demographic and Health Surveys
FCHVs: female community health volunteers
GDP: gross domestic product
MoHP: Nepal’s Ministry of Health and Population
NGOs: nongovernmental organizations
NPR: Nepalese rupees
PPP: purchasing power parity
SSC: skin-to-skin contact
UNICEF: United Nations Children’s Fund
US$: United States of America dollar
VDCs: village development committees
WHO: World Health Organization
